# Prediction of lateral pelvic lymph node metastasis in patients with locally advanced rectal cancer with preoperative chemoradiotherapy: Focus on MR imaging findings

**DOI:** 10.1371/journal.pone.0195815

**Published:** 2018-04-12

**Authors:** Min Ju Kim, Bo Yun Hur, Eun Sun Lee, Boram Park, Jungnam Joo, Min Jung Kim, Sung Chan Park, Ji Yeon Baek, Hee Jin Chang, Dae Yong Kim, Jae Hwan Oh

**Affiliations:** 1 Center for Colorectal Cancer, Research Institute and Hospital, National Cancer Center, Goyang-si, Republic of Korea; 2 Department of Radiology, Chung-Ang University Hospital, Seoul, Republic of Korea; 3 Biometric Research Branch, Research Institute and Hospital, National Cancer Center, Goyang-si, Republic of Korea; University of Texas MD Anderson Cancer Center, UNITED STATES

## Abstract

**Purpose:**

To investigate the predictive factors for lateral pelvic lymph node (LPLN) metastasis in patients with locally advanced rectal cancer treated with preoperative chemoradiotherapy (CRT).

**Materials and methods:**

Fifty-seven patients with locally advanced rectal cancer and LPLNs larger than 5 mm underwent LPLN dissection (LPLD) after preoperative CRT. The MRI findings, including the apparent diffusion coefficient value and LPLN size reduction rate before/after CRT; clinical factors; and pathologic results were evaluated to identify the predictive factors associated with LPLN metastasis.

**Results:**

LPLN metastasis was confirmed in 23 patients (40.4%). Metastasis was significantly higher in LPLNs with multiplicity, short-axis diameter ≥8 mm before CRT, short-axis diameter >5 mm after CRT, size reduction rate ≤33.3%, heterogeneous signal intensity, and irregular margin (P<0.05) on MR. Multivariable analysis showed that pre-CRT short-axis diameter of LPLNs ≥8 mm, size reduction rate ≤33.3%, and heterogeneous signal intensity were independently associated with LPLN metastasis.

**Conclusions:**

The size and signal intensity of LPLN before and after CRT are useful MRI findings to predict LPLN metastasis and are helpful to determine the indications for LPLD.

## Introduction

The combination of preoperative chemoradiotherapy (CRT) and total mesorectal excision (TME) has become the standard treatment for locally advanced rectal cancer, reduced locoregional recurrence, and improved survival rates [1–5]. Nevertheless, there are still cases of locoregional recurrence, which is a significant clinical problem that is associated with severe morbidity, a low likelihood of salvage, and eventually, death. Local recurrence can develop even in the absence of circumferential resection margin involvement, which may be explained by extramesorectal lateral pelvic lymph node (LPLN) metastasis that could not be resected using conventional TME [6]. In addition, some authors have reported that LPLN metastasis is a risk factor of locoregional recurrence, and more than 40% of local recurrences in patients with LPLN metastasis develop without distant metastasis [7–9]. Based on these results, some researchers have proposed surgical treatments to prevent LPLN metastasis, such as lateral pelvic lymph node dissection (LPLD) [10, 11]. However, routine adoption of LPLD may not be beneficial for all patients with locally advanced rectal cancer because only 10–23% of patients with locally advanced rectal cancer have LPLN metastasis [6, 12], and LPLD could lead to complications, such as impaired urinary and sexual function, resulting in poor quality of life [13–15]. Therefore, it is essential to identify the preoperative predictive factors of LPLN metastasis and to identify the optimal indication for LPLD in patients with locally advanced rectal cancer treated with preoperative CRT.

Magnetic resonance imaging (MRI) is assumed to be an optimal diagnostic modality for tumor staging in rectal cancer due to its high soft-tissue contrast [16–18]. However, there is a wide-ranging accuracy of 62–85% and a relatively poor sensitivity for lymph node staging [18–22]. Recently, some reports have suggested the diagnostic criteria to determine metastatic lymph nodes to be the margin, signal intensity and size [20, 22, 23]. Additionally, several studies have reported positive results to determine metastatic lymph nodes in head and neck cancer [24, 25], uterine cervical cancer [26, 27], and rectal cancer [21, 28] using diffusion-weighted imaging (DWI), which is an emerging functional imaging technique that is used in oncological applications, such as tumor detection, characterization and response evaluation. However, the results revealed the diagnostic performance of only mesorectal lymph nodes [18, 21, 22, 28]. Additionally, some previous studies concerning metastatic LPLNs focused on the size criteria or clinicopathologic findings [8, 29]. Thus, we expected that the diagnostic performance for predicting LPLN metastasis would be improved by adding the margin, signal intensity, and DWI in addition to the size and preoperative clinical findings. To our knowledge, few studies have evaluated these preoperative MRI findings, including DWI for predicting LPLN metastasis in locally advanced rectal cancer. In addition, we were interested in changes in the MRI findings before and after preoperative CRT.

Therefore, the purpose of this study was to investigate the predictive factors for LPLN metastasis in patients with locally advanced rectal cancer treated with preoperative CRT, focusing on MRI findings with DWI.

## Materials and methods

### Patients

The Institutional Review Board of National Cancer Center, Korea, approved this study (NCC2015-0162) and waived the requirement for informed patient consent because we retrospectively analyzed data. One-hundred-seven patients with primary rectal cancer underwent LPLD at our institute between September 2011 and April 2015. LPLD was performed for patients with LPLNs ≥ 5 mm in the short-axis diameter as seen on MRI. In cases in which preoperative CRT was performed, LPLNs ≥ 5 mm in the short-axis diameter before CRT were indicated for LPLD, regardless of the imaging findings after CRT. The inclusion criteria of this study were as follows: (1) histologically proven rectal adenocarcinoma of the middle or distal rectum (within 10 cm above the anal verge), (2) locally advanced resectable disease (stage II or III) under clinical examination using rectal MRI or pathologic examination, (3) curative surgical resection after preoperative CRT, and (4) no previous or concurrent other malignancy. We excluded patients who had undergone salvage operations (n = 10), had not undergone CRT (n = 31), or radiation therapy (n = 5), or had a confirmed neuroendocrine tumor (n = 1). Of the 60 eligible patients, two underwent delayed operation and one had no available pre-CRT MRI. We analyzed the remaining 57 patients.

As previously described [8], before preoperative CRT, staging workups were performed on all patients. These workups included a digital rectal examination, a complete blood count, liver function tests, serum measurements of carcinoembryonic antigen (CEA), video colonoscopy, chest computed tomography (CT), abdominal and pelvic CT, and rectal MRI with or without transrectal ultrasonography. To evaluate the response to preoperative CRT, rectal MRI was performed prior to surgery with the same MR protocol as in the initial workup.

### Treatment

The preoperative CRT protocol was described in our previous report [8]. Radiotherapy was delivered to the entire pelvis with a dose of 45 Gy in 25 fractions, followed by a boost of 5.4 Gy in 3 fractions to the primary tumor within 6 weeks. The lateral pelvic area was usually included in the radiation target volume. Chemotherapy was administered concurrently with radiotherapy in all patients, and one of the following chemotherapeutic regimens was used: 5-fluorouracil and leucovorin (n = 17); capecitabine (n = 36), capecitabine and oxaliplatin (n = 2); or others (n = 2). After the completion of preoperative CRT, all patients underwent curative resection, including TME, high ligation of the inferior mesenteric vessels and LPLD. The median interval between CRT and surgery was 6 weeks (range, 4–8 weeks), and sphincter-preserving surgery was undertaken in 52 (91.2%) patients. Unilateral LPLD was performed on 40 (70.2%) patients, and bilateral LPLD was performed on 17 (29.8%) patients according to the pre-CRT rectal MRI findings.

### Pathologic findings

After radical surgery, all tumor specimens were reviewed by an experienced pathologist, and all of the tumor and mesorectal fat was serially sliced into 4-mm-thick sections and embedded in paraffin. The post-CRT pathologic stage (ypT and ypN) was determined using the American Joint Committee on Cancer (AJCC) Seventh Edition Staging System. The tumor regression grade (TRG) was microscopically evaluated using the scale of the modified Dworak (mDworak) TRG system [30]. Regression was graded as follows: TRG 4 = complete regression, defined as no residual tumor cells in the primary tumor and regional LNs (ypT0N0); TRG 3 = near complete regression, defined as one or two microscopic foci of residual tumor cells or groups in the primary tumor and regional LNs; TRG 2 = moderate regression, defined as dominant fibroinflammatory changes with vasculopathy encompassing more than 50% of the entire tumor, including the tumor, regional LN metastases, and perirectal tumor deposits; and TRG 1 = minimal regression, defined as a dominant tumor mass encompassing more than 50% of the primary tumor and/or regional LN metastases. Table 1 shows the distribution of the ypT and ypN classification and mDworak TRG. The total number of harvested lymph nodes and number of metastatic lymph nodes in each region were recorded. Histopathologic evaluation of LPLN was the diagnostic standard.

**Table 1:** Patient characteristics.

			LPLN metastasis			
		Total	Negative	Positive	P value	
		(N = 57)	(N = 34)	(N = 23)		
Age (years)	median (IQR)	57 (50–67)	53.5(50–62)	64 (51–67)	0.179	*
Gender						
	Female	24	16	8	0.357	†
	Male	33	18	15		
AV (cm)	median (IQR)	5 (3–6)	5 (4–7)	4 (3–6)	0.302	*
	≤5	33	18	15	0.357	†
	>5	24	16	8		
Tumor size (cm)	median (IQR)	5 (4–6)	5 (5–6)	5 (4–6)	0.303	*
	≤5	39	23	16	0.879	†
	>5	18	11	7		
Pretreatment CEA (ng/mL) (miss = 2)	median (IQR)	4.3 (1.9–12.8)	4.3 (2.1–11.6)	5.8 (1.8–16.8)	0.898	*
	≤5	29	19	10	0.378	†
	>5	26	14	12		
Histological grade						
Low (well/moderate)		53	32	21	0.683	†
High (Mucinous/poor/signet)		4	2	2		
cT						
	2	1	1	0	0.130	‡
	3	45	24	21		
	4	11	9	2		
cN						
	Negative	0	0	0	1	‡
	Positive	56	34	23		
ypT						
	0	5	4	1	0.754	‡
	1	1	1	0		
	2	9	5	4		
	3	35	21	14		
	4	7	3	4		
ypN						
	0	21	21	0	< .0001	†
	1	19	9	10		
	2	17	4	13		
Tumor regression grade						
	<2	19	10	9	0.445	†
	≥2	38	24	14		
Venous invasion						
	Negative	36	24	12	0.157	†
	Positive	21	10	11		
Angiolymphatic invasion						
	Negative	32	23	9	0.033	†
	Positive	25	11	14		
Perineural invasion						
	Negative	33	22	11	0.205	†
	Positive	24	12	12		
Preoperative chemotherapy					0.152	†
	5-fluorouracil and leucovorin	17	13	4		
	capecitabine	36	20	16		
	capecitabine and oxaliplatin	2	1	1		
	others	2	2	0		
Surgery					0.348	†
	Sphincter preserving	52	32	20		
	Abdominoperineal resection	5	2	3		

Note.—IQR, interquartile range; AV, distance from anal verge; CEA, carcinoembryonic antigen; LPLN, lateral pelvic lymph node; CRT, chemoradiotherapy

* Wilcoxon rank-sum test

† Chi-sqaure test

‡ Fisher's exact test

### MR protocol and analysis

All patients underwent rectal MRI using either one of three 3T superconducting systems (Achieva 3.0T and Achieva 3.0T TX, Philips Healthcare, Cleveland, OH, USA; and Signa HDX 3.0T, GE Healthcare, Milwaukee, WI, USA) with pelvic phased-array coils before and after CRT prior to surgery. Axial, sagittal and coronal T2-weighted fast spin-echo acquisitions in the pelvis were obtained using a 25-cm field of view, 3-mm section thickness, 1-mm intersection gap, repetition time (ms)/echo time (ms) of 3000–4000/65–90, 320×256 matrix, echo-train length of 21, and no fat saturation. Axial images perpendicular to the long-axis of the rectum with a 4-mm section thickness were also obtained using a 20-cm field of view. DWI was also obtained using a single-shot echo-planar imaging sequence. The same parameters were used to match the tumor on axial T2-weighted images. To obtain a high b-value DWI, we used b factors of 0 and 1000 sec/mm^2^. It was not necessary for patients to hold their breath during DWI. The apparent diffusion coefficient (ADC) values (×10^3^ mm^2^/s) were calculated as follows: ADC = In(S0/S1)/(b1-b0).

All MR images were retrospectively reviewed by two gastrointestinal radiologists who were blinded to the patients’ clinical and pathologic information. Differences in the radiologic conclusions were resolved by consensus. When LPLN was detected as ≥ 5 mm in the short-axis diameter on pre-CRT MRI, we included the patients in this study. We evaluated all visible LPLNs in MRI scans, even those with short-axis diameters smaller than 5 mm. The following LPLN characteristics were analyzed on pre- and post-CRT MRI: location, short-axis diameter, signal intensity, margin, DWI signal intensity, and ADC value. The location of LPLN was grouped into six regions according to anatomic landmarks: both external iliac, both obturator and both internal iliac areas. After measurement of the short-axis diameters of MRI-detected LPLNs on pre- and post-CRT MRI, a size reduction in the percentage of LPLNs was calculated. The size reduction rate of LPLN was defined as (Dpre–Dpost / Dpre) × 100%, where Dpre and Dpost are the short-axis diameters of the LPLNs before and after CRT, respectively [31]. The post-CRT LPLN response was defined as persistent when the short-axis diameter was >5 mm and as responsive when the short-axis diameter was ≤5 mm. The signal intensity was categorized as homogeneous or heterogeneous, and the margin was categorized as well defined or irregular. The DWI signal intensity was classified as high, iso, and low relative to that of adjacent muscle. To obtain the ADC values, a free-hand region of interest (ROI) was placed on the ADC map as large as possible within the LPLN. The DWI and ADC map were not available for all patients, and further analysis was performed using only the available data. The characteristics of LPLNs were not assessed on post-CRT MRI because of the difficulties posed by LPLN shrinkage due to CRT.

An MRI-detected LPLN was regarded as true-positive when the region was positive for a metastatic node in the pathologic examination. In the case of more than one LPLN on MRI, the nodes were all regarded as true-positive unless the number of nodes on MRI exceeded the number of pathologically determined positive nodes. When the number of LPLNs on MRI exceeded the number of pathologically determined positive nodes, we regarded the larger nodes on MRI as positive. LPLNs on MRI were regarded as false-positive when the region was negative according to the pathologic examination [32].

CT scans were performed on all patients on postoperative day 7. We compared preoperative and postoperative CT scans to evaluate whether the MRI-detected LPLNs had been removed.

### Statistical analysis

For statistical analysis, we selected the largest LPLN per patient if multiple LPLNs were detected on pre-CRT MRI. The distribution of data was presented as median (IQR) for continuous variables and frequency (percentage) for categorical variables. To compare the clinicopathologic findings according to LPLN metastasis, age, gender (male vs female), distance from the anal verge (≤5 vs >5 cm), tumor size (≤5 vs >5 cm), pretreatment CEA level (≤5 vs >5 ng/mL), histologic grade (low vs high), cT classification, cN classification, ypT classification, ypN classification, tumor regression grade (<grade 2 vs ≥grade 2), and pathological results of venous, angiolymphatic, and perineural invasion were considered. To determine the predictive factors for LPLN metastasis, Pearson’s chi-square test or Fisher’s exact test was used for categorical variables, and Wilcoxon rank-sum test was used for continuous variables.

Regarding MR imaging findings to predict LPLN metastasis, the number of MRI-detected LPLNs (1 vs ≥2), short-axis diameter before CRT (<8 vs ≥8 mm)/after CRT (≤5 vs >5 mm), size reduction rate (≤33.3 vs >33.3%), signal intensity before CRT (homogeneous vs heterogeneous), margin before CRT (well-defined vs irregular), DWI signal intensity before CRT (low or iso vs high), and ADC value before CRT were analyzed in univariable analysis. The cutoff values of short-axis diameter before/after CRT and size reduction rate were determined based on the results of ROC analysis. Receiver operating characteristic (ROC) analysis was performed to determine the optimal cutoff values of the short-axis diameters before/after CRT, as well as the ADC values of LPLNs before CRT, to predict LPLN metastasis. The optimal cutoff point was defined as the value at which the sum of the sensitivity and specificity was maximized.

Among the post-CRT MRI findings, only the short-axis diameter was included for statistical analysis because the others were not significantly different from the pre-CRT MRI findings. The pre- and post-CRT MRI findings were different in only five patients according to the signal intensity, eleven patients according to the margin and thirteen patients according to the DWI signal intensity of LPLN. Some reports have stated that it is difficult to differentiate a metastatic lymph node from a lymph node with irradiation changes on post-CRT MRI using morphological criteria [17, 33, 34]. Thus, the pre-CRT MRI findings were used and the post-CRT MRI findings were excluded from this study.

Multivariable analysis was performed using a logistic regression model with a backward selection method containing all variables that attained univariable statistical significance (P<0.05), but pathological variables were excluded because this study searched for preoperative predictive factors for LPLN metastasis. We also excluded the short-axis diameter after CRT in multivariable analysis because the LPLN size after CRT was not an independent value and is closely related to the size before CRT.

All statistical analyses were performed using commercially available software (SPSS, version 19.0; SPSS, Chicago, Ill). A P value less than 0.05 was considered to indicate statistical significance.

## Results

### Patient characteristics and analysis of clinicopathologic factors

The patient characteristics and associations between clinicopathologic factors and LPLN metastasis are shown in Table 1. As expected, the ypN classification and pathologic angiolymphatic invasion were significantly associated with LPLN metastasis. Other clinical or pathologic parameters were not associated with LPLN metastasis.

The median time interval between post-CRT MRI and surgery was 2 days (range:1–28 days). In total, 608 LPLNs were surgically removed, and the mean number of harvested LPLNs was 11 (range:1–34). LPLN metastasis was pathologically identified in 46 LPLNs from 23 patients. Pre-CRT MRI scans revealed 114 LPLNs, with single LPLNs being detected in 27 (47.4%) patients and multiple LPLNs being detected in 30 (52.6%) patients. Four MRI-detected LPLNs were not surgically dissected, but the other 110 MRI-detected LPLNs were surgically removed LPLNs and were pathologically evaluated. Of the 110 MRI-detected and pathologically evaluated LPLNs, LPLN metastasis was confirmed in 31 LPLNs (28.2%) from 23 patients (40.3%). Nine patients showed LPLN metastasis without mesorectal lymph node metastasis.

### Analysis of MRI findings of LPLNs

The median short-axis diameter of LPLNs was 8 mm (range: 5–21 mm) before CRT and 5 mm (range: 0–17 mm) after CRT. There were significant differences in the median short-axis diameters of the LPLNs before and after CRT between the LPLN metastasis-positive and -negative groups. The median short-axis diameter before CRT was 11 mm (range: 6–21 mm) and was 7 mm (range: 5–13 mm) in patients with and without LPLN metastasis, respectively (P<0.0001), and the median short-axis diameter after CRT was 8 mm (range: 3–17 mm) and 4 mm (range: 0–7 mm) in patients with and without LPLN metastasis, respectively (P<0.0001). There were 34 persistent LPLNs and 76 responsive LPLNs among the 110 MRI-detected LPLNs. Of 34 persistent LPLNs, 25 (73.5%) LPLNs were positive nodes, whereas only six malignant LPLNs were found in 76 responsive LPLNs, which was significantly different (P<0.0001). In terms of the ADC value before CRT, the median ADC value was not significantly different between the two groups (P = 0.617, Table 2).

**Table 2:** MR imaging findings of LPLN.

				LPLN metastasis			
			Total	Negative	Positive	P value	
			(N = 57)	(N = 34)	(N = 23)		
No. of MRI-detected LPLN							
	1		27	21	6	0.008	†
	≥2		30	13	17		
Short diameter before CRT (mm)							
	median (IQR)		8 (7–10)	7 (6–8)	11 (8–13)	< .0001	*
	<8		26	23	3	< .0001	†
	≥8		31	11	20		
Short diameter after CRT (mm)							
	median (IQR)		5 (4–7)	4 (3–5)	8 (6–11)	< .0001	*
	≤5(responsive)		33	30	3	< .0001	†
	>5(persistent)		24	4	20		
Size reduction (%)							
	≤33.3		34	16	18	0.018	†
	>33.3		23	18	5		
Signal intensity before CRT							
	Homogeneous		27	25	2	< .0001	†
	Heterogeneous		30	9	21		
Margin before CRT							
	Well-defined		35	27	8	0.001	†
	Irregular		22	7	15		
DWI before CRT (miss = 3)							
	Low/Iso		1	1	0	0.403	†
	High		53	31	22		
ADC value before CRT (×10^-3^mm^2^/s) (miss = 6)							
	median (IQR)		0.88(0.80–1.16)	0.87(0.78–1.17)	0.92(0.87–1.04)	0.617	*

Note.—LPLN, lateral pelvic lymph node; CRT, chemoradiotherapy; IQR, interquartile range; DWI, diffusion-weighted imaging; ADC, apparent diffusion coefficient

*Wilcoxon rank-sum test

†Chi-sqaure test

The cutoff value of the short-axis diameter of the LPLNs before CRT was 7.5 mm based on ROC analysis to determine the optimal point for the prediction of LPLN metastasis. The area under the ROC curve (AUC) was 0.86, with a sensitivity of 87.1% and specificity of 69.6%. When the cutoff value of the short-axis diameter of the LPLNs after CRT was set at 5.5 mm, an AUC of 0.916, sensitivity of 80.6%, and specificity of 87.3% were obtained to predict LPLN metastasis.

Regarding univariable analysis of the MR imaging findings (Table 2), the multiplicity of MRI-detected LPLNs, short-axis diameter before and after CRT, size reduction rate, signal intensity before CRT, and margin before CRT were significantly associated with LPLN metastasis. Metastasis was significantly higher in LPLNs with multiplicity, pre-CRT short-axis diameter ≥8 mm, post-CRT short-axis diameter >5 mm, size reduction rate ≤33.3%, heterogeneous signal intensity, and irregular margin (P<0.05). The DWI signal intensity and ADC values were not associated with LPLN metastasis.

### Multivariable analysis

Multivariable analysis (Table 3) showed that a pre-CRT short-axis diameter ≥8 mm, size reduction rate ≤33.3%, and heterogeneous signal intensity of LPLN were significantly associated with LPLN metastasis (P<0.05). The odds ratio (OR) of the pre-CRT short-axis diameter of the LPLN ≥8 mm was 7.38 (95% CI, 1.06–51.32), and that of the size reduction rate ≤33.3% was 10.16 (95% CI, 1.70–60.67). The OR of the heterogeneous signal intensity of the LPLN was 16.91 (95% CI, 2.36–121.08). On the other hand, the multiplicity of MRI-detected LPLNs and margins before CRT were not associated with LPLN metastasis on multivariable analysis.

**Table 3:** The multivariable analysis using logistic regression model.

		OR (95% CI)	P-value
No. of MRI-detected LPLN			0.811
	1		
	≥2		
SD before CRT (mm)			0.043
	<8	1	
	≥8	7.38 (1.06–51.32)	
Size reduction (%)			0.011
	≤33.3	10.16 (1.70–60.67)	
	>33.33	1	
SI before CRT			0.005
	Homogeneous	1	
	Heterogeneous	16.91 (2.36–121.08)	
Margin before CRT			0.188
	Well-defined		
	Irregular		

Backward selection method with alpha 0.05

Note.—LPLN, lateral pelvic lymph node; SD, short diameter; CRT, chemoradiotherapy; SI, signal intensity; OR, odds ratio; CI, confidence interval

## Discussion

This study showed that short-axis diameters ≥8 mm before CRT, size reduction rate ≤33.3%, and heterogeneous signal intensity of LPLN were independently associated with LPLN metastasis in patients with locally advanced rectal cancer with preoperative CRT. In addition, the multiplicity of MRI-detectable LPLN, persistent LPLN, and margin of LPLN before CRT were associated with LPLN metastasis in univariable analysis.

These MRI findings, such as short-axis diameters, size reduction rate, and signal intensity, would be helpful to predict LPLN metastasis and select patients who need LPLD preoperatively, although there was the limitation of suggesting a specific cutoff value of the diagnostic criteria due to the small sample size. Some studies [8, 35, 36] have suggested that LPLN recurrence is a major site of local recurrence in patients with locally advanced rectal cancer treated with a combination of curative resection and CRT. In this regard, the preoperative prediction and selection of patients with LPLN metastasis are essential because LPLD for the selected patients could have the potential to improve locoregional control.

We also performed multivariable analysis regarding all 110 MRI-detected LPLNs by using a mixed model containing same variables of the logistic regression model. The mixed model considering all of LPLNs showed almost the same results as those of the logistic regression model considering the largest representative LPLN. Therefore, the MRI findings of the largest representative LPLN could be used as a surrogate marker to determine the indications for LPLD.

LPLN metastases in our study were identified in 23 of 57 patients. The incidence of LPLN metastasis of our study population was 40.3%, relatively higher than that of previous studies [6, 12]. The reason for this high incidence may be that LPLD was performed only for patients with LPLN ≥5 mm in the short-axis diameter. Considering that the possible complications of LPLD result in poor quality of life and that LPLD should be performed selectively for suitable patients, the high incidence of LPLN metastasis of this study is desirable.

Recently, MRI has become a key diagnostic imaging modality for the preoperative evaluation of rectal cancer. Although the diagnostic accuracy of MRI is known to be superior to that of CT for evaluation of the depth of tumor invasion, the accuracy of the nodal status of MRI has been reported to be less reliable than that of local tumor staging [16, 30, 37]. Additionally, no consensus has been reached regarding the size criteria to predict metastatic lymph nodes. Nevertheless, in the diagnosis of metastatic LPLNs using MRI, the lymph node size seems to still be the most reliable parameter. In this study, the short diameter of LPLN was one of the most significant and independent predictors of LPLN metastasis, regardless of CRT. Short-axis diameters ≥8 mm before CRT and >5 mm after CRT were significantly associated with LPLN metastasis. Consistent with these findings, Takashi et al. showed that a short diameter of LPLN ≥8 mm before CRT was independently associated with LPLN metastasis [31], and Oh et al. reported that persistent LPLN on post-CRT was a significant and independent risk factor for LPN metastasis [38] in multivariable analysis. Actually, our study did not include the short-axis diameter after CRT of LPLNs in multivariable analysis because the statistical effect of the short diameter after CRT would be decreased due to its association with the short diameter before CRT. However, the result that the size reduction rate of LPLNs remained as an independently significant predictor of LPLN metastasis indicates that persistent LPLNs were an important parameter for LPLN metastasis. In addition, Oh et al. showed that the recurrence rate was higher and the 5-year survival rate was lower in patients with persistent LPLNs than in patients with responsive LPLNs [38]. Therefore, the indication for LPLD could be based on the size of the LPLNs on MRI before and after CRT for locally advanced rectal cancer.

In the present study, heterogeneous signal intensity was also an independent significant variable to predict LPLN metastasis, whereas an irregular margin was not included as a significant variable according to multivariable analysis, although it showed a significant association with LPLN metastasis in univariable analysis. Recently, some studies [22, 23, 39, 40] emphasized the importance of the signal intensity and border characteristics of lymph nodes on MRI in predicting lymph node metastasis in addition to the size. They demonstrated that the sensitivity and specificity were improved with the additional use of the signal intensity and border relative to size alone to detect metastatic lymph nodes in rectal cancer. Considering the overlap of the size between metastatic and non-metastatic lymph nodes, the addition of the signal intensity and border to the size criteria would increase the diagnostic confidence and accuracy in the prediction of LPLN metastasis.

Several studies have shown the potential value of DWI in the detection of colorectal cancer [41, 42] and assessment of the tumor response to CRT [43, 44]. In addition, several recent studies [21, 45–47] have reported that DWI with ADC values is feasible for differentiating metastatic lymph nodes from benign lymph nodes and that the ADC values of metastatic lymph nodes were significantly lower than those of non-metastatic lymph nodes in the case of rectal [21], breast [45], gastric [46], and endometrial [47] cancer. Based on these studies, we expected that DWI with ADC values could be a useful parameter in detecting LPLN metastasis in locally advanced rectal cancer; however, there was no significant difference regarding the DWI signal intensity and ADC value between LPLN metastasis-positive and -negative groups before and even after CRT. The analysis regarding all 110 MRI-detected LPLNs also showed the same results, and most lymph nodes showed high signal intensity on DWI regardless of metastasis and CRT. Similarly, Roy et al. reported no significant difference between the median ADC value of metastatic LNs and non-metastatic LNs in the pelvic LNs of gynecological malignancies [48]. Additionally, Zhou et al. reported that the sensitivity and negative predictive values for DWI were 100%, with an accuracy for DWI of only 40.4%, and they argued that DWI is currently unlikely to be useful in clinical practice due to its low accuracy [49]. There still remains controversy, and further investigation is needed regarding the application of the DWI and ADC values for discriminating between metastatic and benign lymph nodes, including LPLNs of advanced rectal cancer.

There are several limitations to our study. First, the sample size was small. Second, we did not evaluate the patients with LPLN short-axis diameters of <5 mm. However, routine adoption of LPLD for all patients with locally advanced rectal cancer is non-ethical, knowing that only 10‒23% of patients have LPLN metastasis and possible complications of LPLD, such as impaired urinary and sexual function. Additionally, our previous study [8] showed that the local recurrence rate was significantly low in patients with LPLNs <5 mm. Based on our previous study [8], LPLD was performed at our institute only for patients with LPLNs ≥5 mm in the short-axis diameter. Therefore, the small sample size and limited results of LPLNs smaller than 5 mm could not be avoided. Third, we did not perform validation study for generalization of this result and analyze the diagnostic performance of the MRI findings using ROC analysis with multiple reviewers. Fourth, we made some assumptions in our node-by-node comparisons, so we might have overlooked the metastaticity of some smaller or undetectable lymph nodes and the non-metastaticity of some larger or detectable lymph nodes. Additionally, we did not evaluate the surgical outcome of LPLD and oncological outcome after LPLD, which are important considerations for decisions regarding surgical and oncological treatments. Further study is needed to confirm the diagnostic performance of the MRI findings of LPLNs and whether LPLD is beneficial in locally advanced rectal cancer with preoperative CRT.

In conclusion, the size and signal intensity of LPLNs before and after CRT are useful MRI findings to predict LPLN metastasis and are helpful to determine the indications for LPLD in patients with locally advanced rectal cancer with preoperative CRT.
